# Gram Negative Bacteria Are Associated with the Early Stages of Necrotizing Enterocolitis

**DOI:** 10.1371/journal.pone.0018084

**Published:** 2011-03-22

**Authors:** Erica M. Carlisle, Valeriy Poroyko, Michael S. Caplan, John A. Alverdy, Donald Liu

**Affiliations:** 1 Department of Surgery, University of Chicago Medical Center, Chicago, Illinois, United States of America; 2 Department of Pediatrics, Northshore University Health System, Chicago, Illinois, United States of America; Columbia University, United States of America

## Abstract

**Introduction:**

Necrotizing enterocolitis (NEC) affects 5–10% of infants born weighing less than 1500 g. Most models of NEC recapitulate late-stage disease with gut necrosis and elevated inflammatory mediators. Evaluation of NEC at earlier, less lethal stages of disease will allow investigation of initial disease triggers and may advance our understanding of temporal relationships between factors implicated in NEC pathogenesis. In this manuscript, we describe our investigation of early NEC and test the hypothesis that bacteria and inflammatory mediators differ between animals with early NEC and disease free animals.

**Methods:**

On DOL7 C3HeB/FeJ pups were fed liquid formula with 1×10^4^ *Streptococcus thoraltensis*, *Serratia marcescens*, and *Pseudomonas aeruginosa* every 3 h. To initiate NEC, pups underwent asphyxia (100% N_2_ for 90 s) and hypothermia (4°C for 10 min) after feeding. Pups were euthanized at 72 h. Intestines were collected for histologic NEC scoring and DNA/RNA extraction. Bacterial populations were identified by 16S rRNA pyrosequencing and principal component analysis (PCA). RNA isolates underwent QRT-PCR for Toll-like Receptor 4 (TLR4) and inducible nitric oxide synthase (iNOS).

**Results:**

Despite histologic, intestinal damage in mice with NEC, no gross necrosis was observed suggesting early disease. QRT-PCR yielded no difference between groups in TLR4 or iNOS mRNA levels. PCA demonstrated relative clustering of microbial communities based on presence or absence of NEC. 16S pyrosequencing demonstrated similar phyla between groups (*Firmicutes* and *Proteobacteria* predominated in all animals). However, the colonic microbiota of animals with NEC had more *Citrobacter* (p<0.01), *Klebsiella* (p<0.05), and *Tatumella* (p<0.05), while that of animals without NEC had more *Streptococcus* (p<0.01) and *Enterococcus* (p<0.01).

**Conclusion:**

*Citrobacter*, *Klebsiella*, and *Tatumella* are associated with NEC. Differential colonic bacteria were identified despite the lack of inflammatory mediator elevation traditionally associated with NEC. This suggests a temporal relationship between bacteria and inflammatory mediators such that alterations in gut microbiota are associated with early NEC, while inflammatory mediator elevation is associated with advanced NEC.

## Introduction

Necrotizing enterocolitis (NEC) is an inflammatory necrosis of the intestine that results in significant morbidity and mortality in 5–10% of infants born weighting less than 1500 grams [1], [2]. Of affected infants, 10–30% will succumb to the disease rendering NEC the leading cause of death in this vulnerable population [2]. It is widely believed that the pathophysiology of NEC is multi-factorial with early enteral feeding, abnormal bacterial colonization, hypoxia/intestinal ischemia due to perinatal stress, and an exaggerated inflammatory mediator response all contributing to the development of epithelial cell injury and a weakened mucosal defense system that fosters the development of NEC [3]. However, despite decades of extensive research designed to uncover the cellular and molecular mechanisms that generate disease, the precise etiology of NEC remains elusive. Further, the temporal relationship between the implicated factors is also quite obscure. Specifically, little is currently known regarding which factors are early insults in NEC pathogenesis and which factors are down-stream markers of advanced disease. Given this relatively limited understanding of the molecular pathophysiology of NEC, current treatment remains primarily supportive. For infants with early stages of disease, oral feedings are held, and broad spectrum antibiotics are administered. If disease progresses to frank intestinal necrosis, exploratory laparotomy and resection of necrotic bowel or insertion of a peritoneal drainage catheter is necessary. Mortality in infants requiring surgical intervention ranges from 20–50% [4], [5].

To enrich our understanding of this highly lethal disease, scientists have spent the past three decades working to develop animals models of NEC [6]. Most models utilize preterm or very young animals, and many rely upon hypoxia/cold stress [7], [8], [9], bacterial inoculation [3], [10], formula feeding, and ischemia/reperfusion [11], [12], [13] to promote the development of NEC. Typically, the overall goal and experimental endpoint in animal models of NEC is to recapitulate the most devastating clinical course possible. With this goal in mind, investigators strive to generate overt gut necrosis, severe histological damage, and marked elevation of inflammatory mediators, such as inducible nitric oxide synthase (iNOS) and Toll-like Receptor 4 (TLR4), which have consistently been shown to be drastically elevated in animals with NEC and in intestinal sections resected from babies requiring bowel resection for necrosis [14], [15].

While such models offer an excellent portrayal of devastating, late-stage NEC, focus upon only such extreme disease states may offer limited advancement of our understanding of early etiologic triggers of disease. Perhaps equally as important as enriching our understanding of NEC by studying models of near-lethal disease may be focusing investigation on experimental animals with more mild, presumably earlier stage NEC. Animal models designed to recapitulate early disease (limited gross necrosis, less histologic damage, and more mild patterns of inflammatory mediator activation) will allow increasingly focused analysis of the initial insults associated with the development of NEC and the potential temporal relationships among factors implicated in NEC etiology. Simply stated, investigation of more mild NEC may allow us to revise the age old question of “which came first: the chicken or the egg?” to instead query “which came first: the altered gut microbiota or the inflammatory mediator cascade?” In addition to gaining valuable insight into the potential chronology of involvement of the factors that promote NEC pathogenesis, such investigation may also reveal new therapeutic targets for focused treatment of NEC early in the clinical course thereby preventing the subsequent initiation of bowel necrosis and heightened inflammatory mediator cascades that confer long term morbidity and mortality in these vulnerable infants.

In this manuscript, we describe our efforts to consistently generate early, less severe NEC in a murine model. The model we selected is a slightly modified version of a well-validated NEC model that utilizes preterm mouse pups delivered by Cesarean section, gavage formula feeding, hypoxia/cold stress, and bacterial inoculation to induce NEC [3]. We elected to substitute 7-day old (7 d) mice for preterm pups due to the strong association between Cesarean section and mortality secondary to issues not related to NEC [16]. Additionally, it has been our experience that newborn pups tend to quickly develop advanced, near-lethal NEC when exposed to this model. We have found that use of 7 d old pups in this well-validated model allows for consistent generation of early NEC thus allowing us to investigate the differences in intestinal microbiota and inflammatory mediator expression between animals with early, less severe NEC as compared to animals without disease.

## Materials and Methods

### Animal model

C3HeB/FeJ mice underwent normal vaginal delivery and remained housed with the mother until day of life 7 (DOL7). On DOL7 pups were relocated to a neonatal incubator and exposed to our experimental feeding protocol. Pups were fed 0.12 ml of liquid puppy formula (Esbilac) containing 1×10^4^ *Streptococcus thoraltensis*, *Serratia marcescens*, and *Pseudomonas aeruginosa* every 3 h via orogastric gavage using a 1.9Fr silastic catheter. To initiate NEC, pups experienced asphyxia (100% N_2_ for 90 s) and hypothermia (4°C for 10 min) following each feeding. Pups were euthanized at 72 h, and the small intestine and colon were collected for subsequent analysis. This study was carried out in strict accordance with the recommendations in the Guide for the Care and Use of Laboratory Animals of the National Institutes of Health. The protocol was approved by the Northshore University Health System Institutional Animal Care and Use Committee (Protocol Number: EH09-523).

### Sample collection and tissue processing

Intestinal samples were used for histologic analysis, RNA isolation, and DNA isolation. For histologic analysis, 1 cm sections of duodenum, jejunum, and ileum from each animal were collected and embedded in OCT for cryosectioning. The entire colon of each animal was flash frozen in liquid nitrogen and stored at −80°C for subsequent RNA and DNA extraction.

### Histology

OCT embedded tissue underwent cryosectioning at 5 µm. In an effort to ensure histologic analysis from tissue along the entire length of the small bowel, 3–4 cross-sections from the duodenum, jejunum, and ileum of each animal were prepared. All sections were stained with hematoxylin and eosin (H&E), and scored by a blinded observer according to a well-validated, published scoring rubric [17]. As is well described in the literature, intact villi received a score of 0, sloughing of epithelial cells at the villous tips was assigned a score of 1, and mid-villous damage was assigned a score of 2. Scores of 3 were assigned to sections with complete villous necrosis, and a score of 4 was given for transmural necrosis. The final score for each animal was determined based upon the highest score observed in any particular specimen. Based on this scoring system, a score of 2 or greater is associated with NEC [17].

### Inflammatory Mediator Analysis: Quantitative real-time reverse-transcription PCR (QRT-PCR)

To determine if the inflammatory mediator elevation typically described in NEC could be identified, we determined the total colonic inducible nitric oxide synthase (iNOS) and Toll-like receptor 4 (TLR4) mRNA levels in pups with and without NEC. RNA was isolated from whole colonic tissue samples using Pure-link RNA Minikit (Invitrogen). RNA levels were evaluated by OD measurement at 260 nm on a Nano-drop 2000 (Thermo Scientific, Wilmington, DE). To ensure RNA quality, only samples with A260/280>1.8 were used in further QRT-PCR analysis. cDNA was prepared by reverse transcription from 1 µg of RNA in a 25 µl reaction. Transcript levels were determined using QRT-PCR with Glyceraldehyde-3-phosphate dehydrogenase (GAPDH) as a housekeeping reference gene. All primers were obtained from ABI TaqMan gene expression assays. Each QRT-PCR reaction contained 0.5 µl of cDNA, 0.5 µl of forward and reverse primers, and 9 µl of Perfecta PCR master mix (Quanta Biosciences). 10 µl reactions were loaded into a 72-well rotor, and QRT-PCR was performed in a Rotor-Gene 3000 (Corbett Robotics, Australia) with the following conditions: 95°C for 10 s, 60°C for 30 s, and 68°C for 40 s. A non-template control was included for all QRT-PCR reactions. CT values were determined using the same background subtraction and threshold values for all reactions. For both TLR4 and iNOS, expression values were calculated as a ratio of CT values of gene of interest to GAPDH. Statistical analysis was preformed using student's t-test (SPSS. Chicago, IL). Differences were considered significant at p<0.05.

### Microbiota Analysis: DNA extraction, temporal temperature gradient gel electrophoresis (TTGE) estimation of microbial diversity, and 16S rRNA pyrosequencing

To ensure direct, intra-animal comparison of 16S rRNA sequencing and QRT-PCR results, total DNA was extracted from colonic tissue of those animals with high quality RNA (A260/280>1.8) (n = 8). Initial treatment with 20% SDS solution (Ambion) and Proteinase K (Ambion) was followed by mechanical bead beating to lyse bacterial cells. Samples then underwent phase separation with Phenol-Choloroform-Isoamyl alcohol (25∶24∶1) (Invitrogen). DNA was precipitated via 10 h incubation at −80°C with glycogen (Ambion), 3M Sodium Acetate pH 5.5 (Ambion), and ethanol. The DNA was then centrifuged at 20,000 g at 4°C for 20 minutes. The pellet was washed twice with 70% ethanol, dried, resuspended in sterile water, and stored at −20°C. DNA integrity was verified by 2% agarose gel electrophoresis.

Temporal temperature gradient gel electrophoresis (TTGE) analysis was then performed as described by Magne et al [18]. Specifically, variable regions 3 and 4 of the bacterial 16S rRNA genes were amplified as described [18]. PCR conditions were as follows: 95°C for 10 min (denaturation and enzyme activation), 30 cycles of 95°C for 1 min (denaturation), 50° for 1 min (annealing), 72°C for 1.5 min (elongation), followed by 72°C for 10 min (final elongation). A non-template control was included for all PCR reactions. The Dcode universal mutation detection system (Bio-Rad, Hercules, CA) was then used for sequence-specific separation of amplicons on a 0.1 cm, 8% polyacrylamide gel (7% glycerol and 1× TBE buffer) with 1.25× TAE (40 mM Tris, 20 mM acetate, 1 mM EDTA, pH 8.0) as the electrophoresis buffer. The system was run at a 20 V for 15 min and then increased to 65 V for 15 hours. The temperature of the system was set to increase by 0.2°C per hour from 66 to 70°C [18]. After completion of the run, the gel was stained with SYBR® green solution (Amresco, Solon, OH) for 15 minutes and scanned using the Molecular Imager PharosFX Plus System (Bio-Rad, Hercules, CA).

16S rRNA pyrosequencing was also performed. PCR amplification of extracted DNA was performed as described by Poroyko et al [19]. Specifically, variable regions 1–4 of the 16S rRNA gene were amplified with the TaKaRa Ex Taq PCR mixture (TAKARA Bio USA, Madison, WI) and bar-coded PCR primers A-788R and B-27F (structure: A-788R CGTATCGCCTCCCTCGCGCCATCAGGGACTACCAGGGTATCTAA and B27F CTATGCGCCTTGCCAGCCCGCTCAG-MID-AGAGTTGATCCTGGCTCAG). PCR was performed under the following conditions: 95°C for 10 min (denaturation and enzyme activation), 30 cycles of 95°C for 1 min (denaturation), 50°C for 1 min (annealing), 72°C for 1.5 min (elongation), followed by 72°C for 10 min (final elongation). A non-template control was included for all PCR reactions. PCR products were purified by AMPure kit (Beckman Coulter) and then underwent sequencing on a GS Titanium 70×75 picotiter plate according to the manufacturer's GS-FLX protocol (Roche Applied Science, Indianapolis, IN). Reads were sorted via barcode with SFF software (Roche Applied Science, Indianapolis, IN). Taxonomy was assigned to sequences longer than 100 bp using the Ribosomal Database Project Classifier tool [19]. To compare overall similarity in microbiota composition between groups, we used mothur software to perform a weighted principal component analysis (PCA) [20]. Additionally, statistical tests for differentially abundant 16S-based taxonomic annotations between populations were performed using Metastats methodology to compute nonparametric p-values [21]. Differences were considered significant at p<0.05.

## Results

### Early NEC can be generated in 7 d old mice

**Figure 1:**
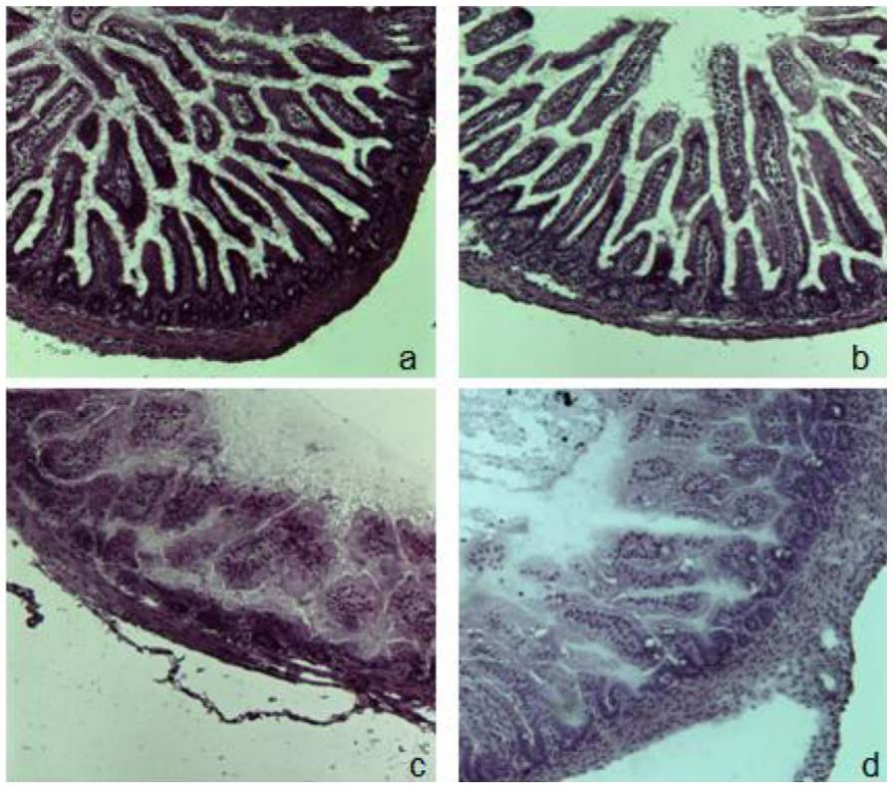
Histology. Representative histology from mice with (c,d) and without (a,b) NEC. After 72 h of exposure to the experimental feeding protocol, intestines were collected and embedded in OCT as described in *Materials and Methods*. Frozen sections were prepared and processed for H&E staining. Sections were scored by a blinded observer based on a well established NEC scoring scale [17].

Twenty-eight mice underwent the experimental protocol. Five mice died prior to the 72 h experimental endpoint presumably due to mechanical issues associated with feeding (aspiration, esophageal perforation, etc.). These animals were not included in the final analysis. Following 72 h exposure to the experimental feeding protocol, bacterial inoculation, and hypoxia/cold stress, no gross intestinal necrosis was observed. However, upon histologic review, NEC (histologic NEC score ≥2) was identified in 9 of 23 mice resulting in a NEC incidence of 39.1%(Figure 1). No animals included in further DNA and RNA analysis received a NEC score higher than 2.

### There is no difference in colonic iNOS or TLR4 mRNA between animals with early NEC and animals without disease

**Figure 2:**
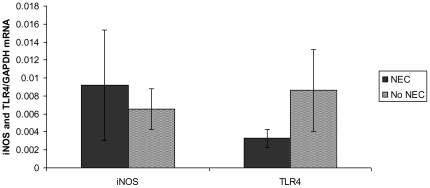
TLR4 and iNOS QRT-PCR Analysis. Colonic TLR4 and iNOS mRNA levels in mice with and without NEC. Following total colonic RNA isolation, cDNA was synthesized, and copy numbers of TLR4 and iNOS were determined using QRT-PCR. Data shown are mean ± SEM of TLR4 and iNOS copy numbers relative to GAPDH. Each column represents n = 4 animals with duplicate measurements.

QRT-PCR demonstrated equivalent TLR4 and iNOS mRNA levels in mice with NEC as compared to mice without NEC (Figure 2).

### Principal component analysis demonstrates clustering based on the presence or absence of NEC

**Figure 3:**
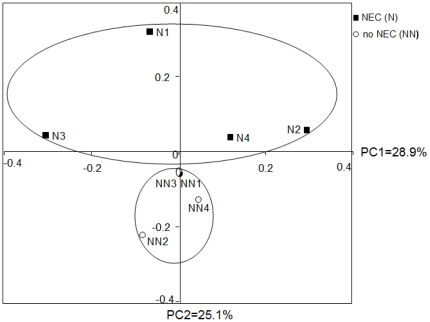
Principal Coordinate Analysis. Results of weighted principal coordinate analysis of the colonic microbiota of mice with NEC (▪N) as compared to mice without NEC (○NN). PC1 = 28.9% and PC2 = 25.1%. This analysis was performed via the mothur analytical pipeline as described for phylotype-based analysis [20].

Weighted principal component analysis demonstrates that mice cluster into relatively distinct groups based on the presence or absence of NEC (Figure 3). Interestingly, while mice without NEC appear to cluster closely together, mice with NEC seem to have a slightly more divergent clustering pattern.

### An increased relative abundance of Gram negative bacteria is present in mice with NEC as compared to mice without NEC

**Figure 4:**
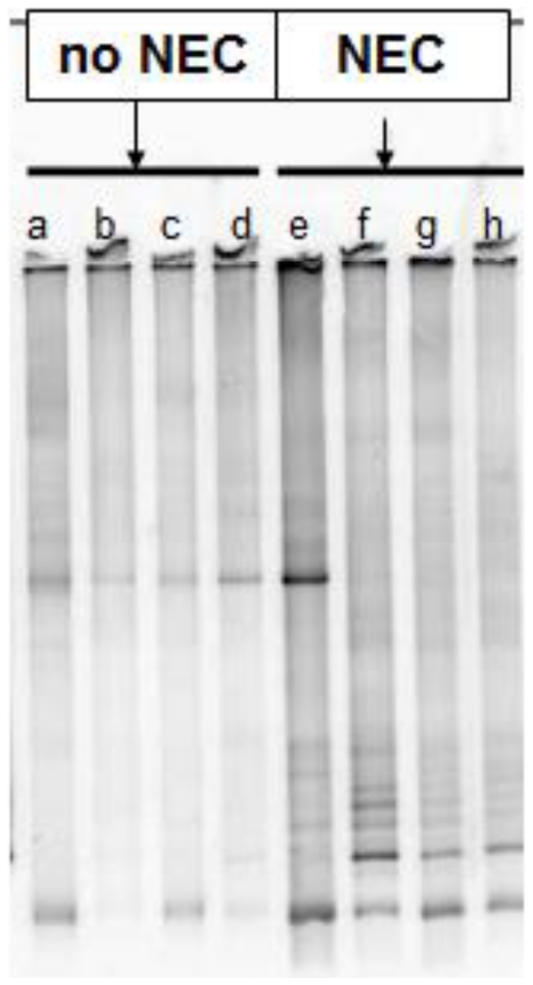
Temporal Temperature Gradient Gel Electrophoresis (TTGE) Analysis. Following total colonic DNA extraction and PCR amplification, PCR products were separated via TTGE to identify bacterial community structures in mice with NEC (e–h) as compared to mice without NEC (a–d).

**Figure 5:**
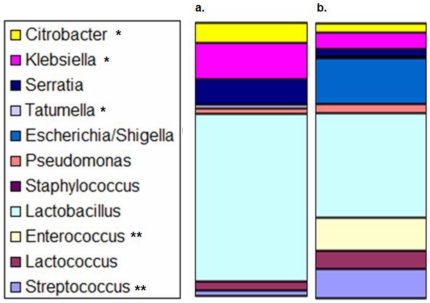
Analysis of Colonic Microbiota. Following total colonic DNA extraction, PCR was performed with 8 bar coded Bact27 and UNIV788 primers to amplify the bacterial 16S rRNA gene. PCR products were purified and underwent subsequent sequencing on the Roche GS-FLX pyrosequencing platform. Taxonomy was assigned to sequences using the Ribosomal Database Project classifier tool. Mice with NEC (a) are compared to mice without NEC (b). Statistical tests for differentially abundant 16S-based taxonomic annotations between populations were performed using Metastats methodology to compute nonparametric p-values. Differences were considered significant at p<0.05. *indicates statistically significantly more in mice with NEC. **indicates statistically significantly more in mice without NEC.

Separation of PCR amplicons on a TTGE gel demonstrated a relatively unique bacterial community fingerprint for mice with NEC as compared to mice without NEC (Figure 4). Pyrosequencing results confirmed a differential bacterial community structure between groups. Specifically, 454 pyrosequencing generated a total of 12,830 reads with an average read-length of 429 bp from 8 libraries. Taxonomic assignments were made for 12,653 sequences. Overall, *Firmicutes* and *Proteobacteria* were the dominant phyla, and *Lactobacillus* was the dominant genus in the colonic microbiota of all animals regardless of disease state. However, *Citrobacter* (p<0.01), *Klebsiella* (p<0.05), and *Tatumella* (p<0.05) (Gram negative organisms, specifically Gamma-proteobacteria from the Enterobacteriaceae family) were associated with the development of NEC, while animals that did not develop NEC had a colonic microbiota with an increased relative abundance of *Streptococcus* (p<0.01) and *Enterococcus* (p<0.01) (Gram positive organisms) (Figure 5).

## Discussion

Over the past three decades, scientists have dedicated tremendous efforts toward creation of animal models of NEC. Pursuit of disease states characterized by gut necrosis, elevated inflammatory mediators, and overt histologic destruction has generated a vast knowledge base regarding the characteristics of late-stage NEC. However, such models offer somewhat limited insight into the early insults necessary for the progression of NEC from a state of relatively mild gut inflammation to a state of true necrosis. To expand our knowledge of the early triggers of NEC we describe our efforts to investigate NEC pathogenesis at an early stage of disease. Despite diagnosis of NEC based on a well-validated histological scoring system [17], no gross necrosis was detected in any of our animals, and no animals received a NEC score higher than 2. This suggests that the disease was in a relatively early stage that preceded the development of gross necrosis and histological destruction characteristic of more advanced disease states (Stage 4). Our subsequent analysis of factors typically implicated in NEC pathogenesis (bacteria and inflammatory mediators) along with our PCA suggests that even at very early stages of disease, a unique gut bacterial community structure exists in animals with NEC as compared to disease-free animals. Given that the mice in our model lacked the inflammatory mediator elevation typical of late-stage NEC, we can extrapolate that the elevation in inflammatory mediators is a later marker of more advanced disease. Taken together, these findings suggest that a temporal relationship may exist between gut microbiota and inflammatory mediators such that changes in gut bacteria may be associated with early NEC pathogenesis while inflammatory mediator elevation may occur later in the progression of disease.

In addition to finding that alterations in gut microbiota are associated with the early stages of NEC, our work utilizes non-culture based technologies to identify specific pathogens that are associated with disease. Recent work demonstrates that only about 5% of all known bacterial species and 30–40% of all bacteria associated with the human body have been successfully cultivated with traditional culture based laboratory techniques [22]. Our utilization of novel molecular approaches to bacterial identification, such as 454 pyrosequencing of the 16S ribosomal subunit, a highly conserved region present within all bacterial cells with a hypervariable region that differs among different bacteria species, thus allows in-depth molecular analysis of organisms present at the time of disease without reliance on traditional culture based techniques. Interestingly, our work demonstrates an increased relative abundance of Gram negative organisms (*Citrobacter*, *Klebsiella*, and *Tatumella*) in the colonic flora of animals with disease as compared to animals without disease. While the finding of increased Gram negative bacteria with NEC has been previously described in studies using traditional culture based technology [23], [24], the molecular mechanisms by which this class of bacteria may be uniquely well-suited to play a role in NEC pathophysiology remain unknown. Further investigation into the properties of Gram negative bacteria that may confer NEC is essential for a thorough understanding of NEC pathophysiology.

However, the mere presence of bacteria in the setting of disease offers limited knowledge regarding the alterations in the bacterial genome that confer increased virulence and subsequent disease progression. Future experiments that employ cutting-edge molecular techniques to explore the metatranscriptome (community-wide bacterial gene expression) of bacteria associated with early NEC pathophysiology will provide novel insight into the bacterial genes called upon to initiate disease. It is also quite possible that alterations in the host mucosal epithelium secondary to anoxia, formula feeding, and other factors manipulated to generate NEC may promote an environment in the gut lumen that fosters a survival advantage for certain bacterial species. Further evaluation of the complex host-microbe relationship on a molecular genetic level will offer key insight into the pathogenesis of NEC.

Continued refinement of our understanding of the early factors of NEC pathophysiology along with increased clinical availability of 16S rRNA sequencing will increase our ability to offer early therapeutic interventions to infants deemed at-risk for NEC. Preliminary applications of the 16S rRNA sequencing techniques utilized in our experimental model are already underway in efforts to enrich our understanding of the microbial ecology of preterm infants. For example, in a study of 23 neonates born at 23–32 weeks gestation, Mshvildadze et al determined that while the overall microbiotic profiles in babies with NEC did not differ from that of controls, babies with NEC had increased levels of *Citrobacter* and *Enterococcus* as compared to babies without NEC [25]. A differential gut microbiota structure between infants with and without NEC was also identified by de la Cochetiere who prospectively analyzed stool samples from 3 infants who developed NEC and 9 who did not to determine that *Clostridium perfringens* was present in babies that developed NEC but not in control infants [26]. Conversely, Millar et al utilized 16S rRNA based sequencing analysis of fecal microbiota of infants and found no difference between babies with and without NEC [27]. As suggested by Young el al in their review of potential biomarkers for NEC, continued utilization of 16S rRNA based sequencing techniques will offer new opportunities to delineate the gut microbial community structures that are conducive to NEC pathogenesis [28]. A richer understanding of these bacterial communities will provide novel insight into how clinicians may be able to manipulate gut microbiota via targeted antibiotics or probiotics to develop prophylactic therapies for NEC [28]. Given, realistic concerns of increased multi-drug resistant bacteria secondary to aggressive antibiotic use, such focused treatment approaches may be preferable to the current practice of empiric administration of broad spectrum antibiotics for suspected NEC. Additionally, transition to prophylactic rather than supportive treatment of NEC would allow clinicians to more accurately and aggressively treat early NEC such that disease progression to advanced disease states warranting surgical intervention or drainage could be avoided.

While we are hopeful that results from this work enhance the understanding of the microbial contribution to NEC pathogenesis and suggest new approaches to targeted therapy, this study is not without limitations. Although our finding that Gram negative organisms have an increased abundance in animals with NEC is supported by the literature [23], [24], the relatively small sample size in our study demands further investigation to assure consistent reproducibility. Specifically, our DNA/RNA analysis was limited in that rather than analyzing complete groups of mice, we evaluated 8 animals. We elected to only include those animals with the highest quality RNA to assure the most accurate results, however, this study would be strengthened by an increased sample size. Given the numerous experimental models of NEC, our results would also be strengthened if they could be replicated in other models. Until consistent replication of our findings are generated, direct clinical applicability is limited. Additionally, our investigation of inflammatory mediator expression may be enhanced by utilizing techniques such as laser capture microscopy to evaluate mRNA expression at the level of the epithelial cell rather than at the whole tissue level. Finally, our work utilizes a 7-day-old animal rather than a newborn or preterm animal which may be more developmentally analogous to infants plagued with NEC. An older animal was utilized given that consistent generation of early disease in preterm or newborn animals is difficult because exposure to the experimental conditions that generate NEC causes many young animals to progress relatively rapidly to more advanced disease states. However, capturing the early progression of NEC in newborn or preterm animals would allow us to more clearly elucidate the impact of microbes on immature gut physiology. Future experiments will be directed toward this goal. Further, use of older animals may have accounted for our slightly lower NEC incidence. Multiple investigators describe a NEC incidence that ranges from 40–60%, however these studies all utilized newborn animals [16], [29], [30]. Although consistent NEC was generated in our animals, 7 d old animals may be somewhat resistant to the damage induced by this protocol. This decreased vulnerability may have accounted for our slightly lower NEC incidence.

In this manuscript, we suggest that investigation of NEC prior to the onset of gross necrosis and a near-lethal disease state will allow novel insight into the mediators of early disease progression. Our results suggest that despite a lack of inflammatory mediator elevation in mice with early NEC, the gut microbial community structure does differ in animals with early disease as compared to animals without disease. This may illustrate a temporal relationship between bacteria and inflammatory mediators such that an altered gut microbiota is associated with early NEC pathogenesis while inflammatory mediators are a later, downstream marker of disease progression. Further studies to examine the complex, bi-directional relationship between host epithelial cells, gut microbes, and immature gut physiology in the setting of early NEC will offer unique insight into NEC etiology and potential novel therapeutic targets to prevent progression of disease from mild inflammation to severe gut necrosis.
